# Skin Prick Test Reactivity to Common Aero and Food Allergens among Children with Allergy

**Published:** 2014-01

**Authors:** Safoora Hosseini, Raheleh Shokouhi Shoormasti, Rozita Akramian, Masoud Movahedi, Mohammad Gharagozlou, Negar Foroughi, Babak Saboury, Anoushiravan Kazemnejad, Maryam Mahlooji Rad, Alireza Mahdaviani, Zahra Pourpak, Mostafa Moin

**Affiliations:** 1 Immunology, Asthma and Allergy Research Institute, Tehran University of Medical Sciences, Tehran, Iran;; 2 Department of Clinical Immunology and Allergy, Children Medical Center, Tehran University of Medical Sciences, Tehran, Iran;; 3 Department of Biostatistics, Faculty of Medicine, Tarbiat Modarres University, Tehran, Iran

**Keywords:** Allergens, Children, Skin test

## Abstract

**Background: **The prevalence of allergic diseases has risen in the last decades. The objective of this study was to determine the common allergens in children via the skin prick test.

**Methods: **This cross-sectional study recruited 313 allergic children (4 months to 18 years old) referred to the Asthma and Allergy Clinic of Children’s Medical Center in Tehran. A questionnaire containing demographic data and patient history was completed. The Skin Prick Test (SPT) was selected according to the patients’ history of food and/or aeroallergen sensitivity.

**Results: **Patients (62.4% male, 37.6% female) with symptoms of asthma (n=141, 57.1%), allergic rhinitis (n=50, 20.4%), atopic dermatitis (n=29, 11.7%), and urticaria (n=20, 8.1%) were studied. Positive skin prick test to at least one allergen was 58.1%. The most prevalent allergens were tree mix (26%), *Alternaria* *alternata* (26%), weed mix (23.6%), *Dermatophagoides farinae* (22.9%), *Dermatophagoides pteronyssinus* (22.9%), milk (21.7%), eggs (20%), and wheat flour (18.3%). Also, common allergens in the patients with different symptoms of allergic disorders were as follows: asthma (tree mix, weed mix, and *Dermatophagoides farinae*); allergic rhinitis (*Dermatophagoides farinae*, tree mix, and *Dermatophagoides pteronyssinus*); and atopic dermatitis (*Alternaria alternata, Dermatophagoides* *pteronyssinus*, and cockroaches).

**Conclusion: **Identifying allergens in each area is necessary and has an important role in the diagnosis and management of allergic disorders and possibility of performing immunotherapy. In this study, the most common aeroallergens were tree mix, *Alternaria alternata*, and weed mix and also the most common food allergens were milk, eggs, and wheat. Considering these data, appropriate preventive strategies can decrease the cost and morbidity of therapeutic actions.

## Introduction

The prevalence of allergic disorders such as atopic dermatitis, allergic respiratory diseases, and food allergy has increased in the last decades and become a major medical concern.^1^^-^^4^ To understand the cause of this trend, researchers have studied such environmental factors as air pollution,^2^^,^^3^^,^^5^ decrease in exposure to microbiologic stimuli,^6^ and changes in dietary habits.^7^

Allergy is an overreaction (immune-mediated reaction) to various proteins (allergens),^8^ first and foremost among which are aeroallergens (e.g. trees, grasses, weeds, moulds, house dust mites, and animal dander).^9^ In addition, other factors known to be responsible for the development of atopy include genetic factors, age of exposure to allergens,^9^ and number of siblings.^10^

For the confirmation of the presence of allergy, the most common diagnostic procedures available are the IgE specific test, food challenge test, and skin prick test (SPT).^8^^,^^11^ The relationship between the results of the SPT and the allergic disease is interpreted by the patient’s clinical history. Allergen selection should be on the basis of the patient’s symptoms, environmental exposures, occupational situation, age, and hobbies and the clinical history of the patient must be considered in the interpretation of the allergy test.^12^ In fact, the SPT is utilized to verify or leave out sensitization to allergens.^13^

The aim of this study was to determine the common allergens in children by means of the SPT results.

## Patients and Methods

The study was performed cross-sectionally on a population of 313 patients with symptoms of allergic disorders who were referred to the Asthma and Allergy Clinic of Children’s Medical Center in Tehran between December 2006 and December 2009 for the confirmation of allergy and appropriate treatment. Adults and patients without clear symptoms were excluded. This study was approved by the Ethics Committee of the Immunology, Asthma, and Allergy Research Institute, Tehran University of Medical Sciences. There is no conflict of interest in this study.

An interview-administered questionnaire containing demographic information, patient history, and family history of allergic diseases was completed. The seasons during which the patients were symptomatic were recorded (4 groups). Consent was verbally obtained from the patients and/or their guardians before the performance of the SPT. The SPT results were later added to the main questionnaire. 

The SPT was selected according to the patients’ history of food and/or aeroallergen sensitivity. The SPT was conducted using a standard allergen extract panel (Stallergenes, France) and comprised histamine and saline respectively as positive and negative controls, 9 aeroallergens (tree mix [maples, horse chestnuts, planes, false acacias, and limes], weed mix [golden rod, dandelion, ox-eye-daisy, and cocklebur], grass mix [cocksfoot, sweet vernal-grass, rye-grass, meadow grass, and timothy], *Dermatophagoides farinae* [DF], *Dermatophagoides pteronyssinus *[DP], cockroaches (Blatella germanica), *Alternaria alternata*, cats, and feather mix [ducks, geese, and hens]), and 6 common food allergens (cow’s milk, eggs, walnuts, hazelnuts, peanuts, and wheat flour).

In the SPT, a small drop of each allergen and control solution is placed on the volar surface of the forearm. In order to avoid false-positive results, the drops must be placed at least 2 cm apart from each other. A needle (25 or 26 gauge) must touch each drop and be penetrated into the epidermal surface at a low angle. The tip of the needle must then be gently lifted up to raise the epidermis, while no bleeding is induced. After about one minute, the solution is wiped away with a cotton tissue. Each test must be performed with separate needles. The SPT shows a reaction which reaches the peak in 15 to 20 minutes for allergens. By means of a millimeter ruler, the largest and smallest diameters of each complete reaction are measured; the result is summed and then divided by 2 (mean diameter). A wheal diameter >3 mm and a flare diameter >10 mm are considered positive results and indicative of clinical allergy.^14^

*Statistics*

The statistical analyses were performed using SPSS (version 15) as well as descriptive statistics and the chi-squared test. A P<0.05 was considered statistically significant.

## Results

**Figure 1:**
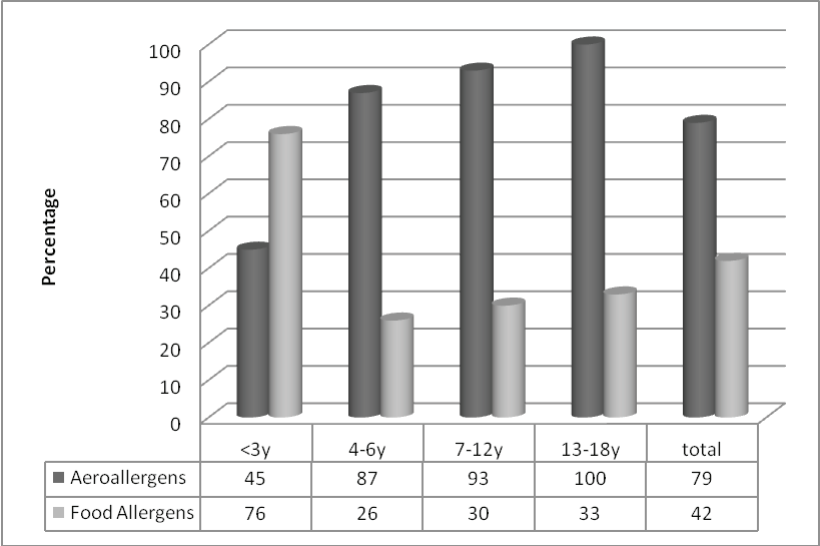
Comparison of sensitivity to aeroallergens and food allergens between different age groups.

A total of 313 subjects, comprised of 118 female (37.6%) and 195 male patients (62.4%) aged between 4 months and 18 years (mean=5.7 years), with asthma symptoms (n=141, 57.1%), allergic rhinitis (n=50, 20.4%), atopic dermatitis (n=29, 11.7%), and urticaria (n=20, 8.1%) were studied. 73 subjects had no definitive diagnosis and were, therefore, considered as missing value. Among them, 58.1% had a positive SPT to at least one allergen. As regards history, 39.1% of the study population had a previous history of allergy and 67% had a positive previous family history of allergy. Most of the subjects were from Tehran (71.6%), Alborz (9.3%), and Mazandaran (2.6%) provinces, and 16.5% were from the other regions of Iran. The most prevalent allergens among the patients were tree mix (26%), *Alternaria alternata* (26%), weed mix (23.6%), DF (22.9%), DP (22.9%), grass mix (21.7%), milk (21.7%), eggs (20%), wheat (18.3%), walnuts (17.1%), hazelnuts (14.9%), and peanuts (14.3%), respectively. Table 1 shows the prevalence of sensitivity to allergens in the different seasons. The patients were divided into 4 groups: 0-3 years (n=111, 35.5%), 4-6 years (n=80, 25.6%), 7-12 years (n=102, 32.6%), and 13-18 years (n=20, 6.4%) (figure 1).

**Table 1:** Prevalence of sensitivity to allergens in different seasons

**Allergens**	**% (N)**	**Spring % (N)**	**Summer % (N)**	**Autumn % (N)**	**Winter % (N)**
Tree mix	26 (43)	34.9 (15)	14 (6)	18.6 (8)	32.6 (14)
Weed mix	23.6 (38)	36.8 (14)	10.5 (4)	18.4 (7)	34.2 (13)
Grass mix	21.7 (38)	39.5 (15)	21.1 (8)	13.2 (5)	26.3 (10)
*Dermatophagoides* * pteronyssinus*	22.9 (43)	30.2 (13)	32.6 (14)	16.3 (7)	20.9 (9)
Dermatophagoides farinae	22.9 (43)	34.9 (15)	23.3 (10)	4.7 (2)	37.2 (16)
Cockroaches	18 (25)	44 (11)	24 (6)	16 (4)	16 (4)
*Alternaria* * alternata*	26 (34)	32.4 (11)	29.4 (10)	14.7 (5)	23.5 (8)
Feather mix	8.4 (10)	20 (2)	0	10 (1)	70 (7)
Cats	15.5 (18)	22.2 (4)	22.2 (4)	22.2 (4)	33.3 (6)
Wheat flour	18.3 (11)	18.2 (2)	0	18.2 (2)	63.6 (7)
Milk	21.7 (20)	15 (3)	25 (5)	15 (3)	45 (9)
Eggs	20 (14)	7.1 (1)	21.4 (3)	21.4 (3)	50 (7)
Walnuts	17.1 (13)	30.8 (4)	7.7 (1)	15.4 (2)	46.2 (6)
Hazelnuts	14.9 (7)	14.3 (1)	28.6 (2)	0	57.1 (4)
Peanuts	14.3 (13)	23.1 (3)	23.1 (3)	15.4 (2)	38.5 (5)

In the spring, the most prevalent allergens were cockroaches (44%), grass mix (39.5%), weed mix (36.8%), and tree mix (34.9%). In the summer, DP (32.6%) and *Alternaria alternata* (29.4%) accounted for the most prevalent allergens. During the autumn, tree mix and weed mix had a prevalence rate of 18.6%, while in the winter, DF (37.2%), weed mix (34.2%), tree mix (32.6%), and feather mix (70%) comprised the most common allergens.

**Figure 2:**
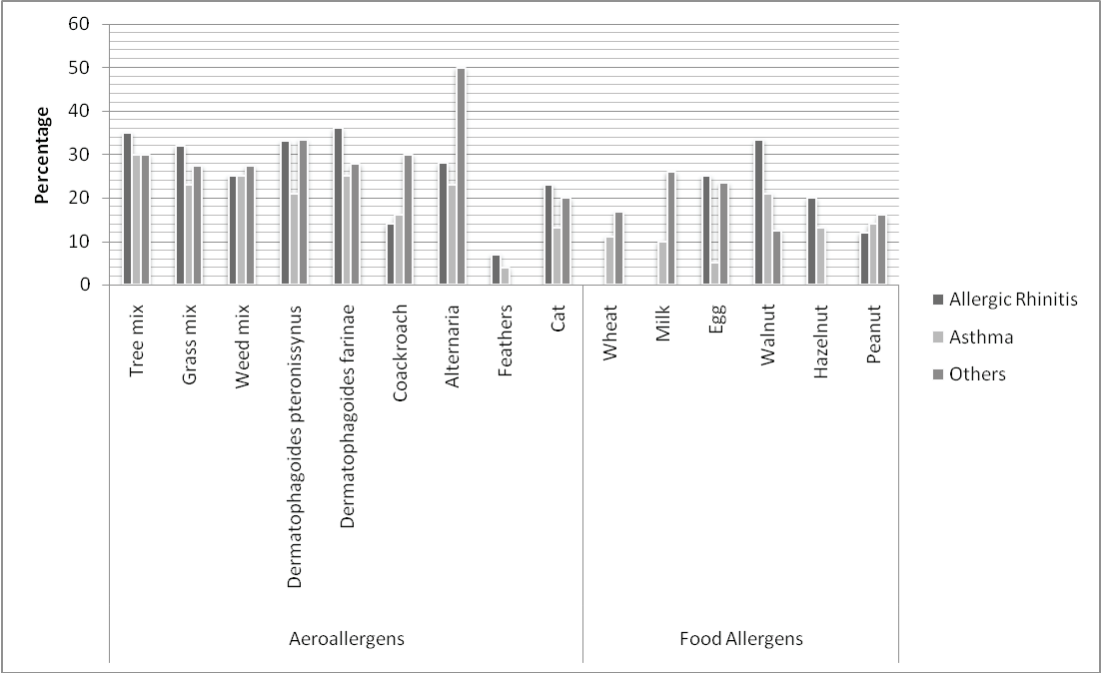
Sensitivity to different allergens according to clinical symptoms. Others: urticaria, and atopic dermatitis

Prevalence of sensitivity to allergens with respect to the clinical symptoms is depicted in table 2 and figure 2. Tree mix, weed mix, and DF, respectively, were the most common allergens in the patients with asthma symptoms, whereas DF, tree mix, and DP, respectively, constituted the most common allergens in the patients with allergic rhinitis. Statistically, there was a significant relationship between sensitivity to food allergens (especially milk and eggs) and aeroallergens in the children <3 and >3 years of age (P<0.01). Among the age groups, the most common allergens were as follows: <3 years (cow’s milk, eggs, hazelnuts, and wheat flour); 4-6 years (*Alternaria alternata*, DF, cat fur, and DP); 7-12 years (grass mix, tree mix, *Alternaria alternata*, and cockroaches); and 13-18 years (weed mix, walnuts, cat fur, and feather mix).

**Table 2:** Prevalence of sensitivity to allergens regarding clinical symptoms

**Clinical symptoms**	**Tree mix % (N)**	**Grass mix % (N)**	**Weed mix % (N)**	**DP % (N)**	**DF % (N)**	***Alternaria*** *** alternate *** **% (N)**
Allergic rhinitis	35 (13)	32 (14)	24.1 (10)	33 (13)	36 (12)	28 (8)
Asthma	30 (29)	23 (23)	25 (23)	21 (19)	25 (24)	23 (17)

DP: *Dermatophagoides pteronyssinus*; DF: *Dermatophagoides farinae*

## Discussion

In this study, 58.1% of a total of 313 subjects showed a positive SPT to at least one allergen. Among them, 57.1% and 20.4% had asthma and allergic rhinitis, respectively. Pollens of trees, grasses, and weeds are the most common allergens that trigger asthma.^15^

In patients with perennial rhinitis and asthma, in whom an extended approach is needed, it is proper to use a chosen panel of outdoor and indoor allergens. These allergens are selected according to both the geographic features and the tree, grass, and weed types as well as the kinds of molds (e.g. *Alternaria alternate*, *Penicillium notatum*, *Aspergillus fumigatus*, and *Cladosporium*), dust mites, cockroaches, and animal dander.^11^

The considerable role of aeroallergens as risk factors for allergic disorders was shown in 141 asthmatic patients in our study, which is in accordance with a similar study on 151 asthmatic patients in Saudi Arabia.^16^ Also, 23.6% of our allergic patients had sensitivity to weeds, which is comparable to a prevalence rate of 21% reported by a study in Zanjan (Iran).^17^ Among our asthmatic patients, positive SPT results for trees, weeds, and grasses were closely similar to the results reported by Farhoudi et al.^18^ and Movahedi and Moin^19^ in Iran. Fereidouni et al.^20^ reported that weeds (81%) and grasses (62%) were the most prevalent allergens in 311 patients with allergic rhinitis. In our asthmatic patients, sensitivity to house dust mites (DP and DF) was 21% and 25%, respectively, which is parallel to the findings by Ceylan et al.^15^ This resemblance could be due to the similar geographical characteristics of the two countries. Our results on the sensitivity to mites and trees are highly in agreement with the findings of Safari et al.^21^ insofar as they reported prevalence rates of 27.3% and 27.2% among patients sensitive to mites and trees respectively; nevertheless, the findings of our two studies are not consistent vis-à-vis the percentages of patients sensitive to grasses (9%) and cockroaches (27.2%).

As was confirmed in our study, pollen levels are usually higher in spring and lower in autumn.^22^ Accordingly, there are various manifestations of allergic symptoms in different seasons. Sensitization to the pollens of trees, grasses, and weeds is higher in spring, and sensitization to house dust mites is elevated in winter for DF and in summer for DP. In contrast, Akarcay et al.^23^ revealed a significant prevalence of sensitization to pollens and house dust mites, both in spring. Overall, the highest prevalence of asthmatic and allergic rhinitis patients suffering from all allergens (aero and food allergens) is seen in winter. It is thought that DF is more frequent in dry climates, whereas DP is more prevalent in humid climates.^23^

Cat fur allergen induces rapid respiratory symptoms in individuals sensitized to cats.^16^ Sensitivity to cat fur allergen was found in 13% of our asthmatic patients. Studies in Iran^18^ and Spain^24^ have reported the prevalence rates of 15% and 15.5%, respectively, but studies in Baltimore^25^and Saudi Arabia^16^ have reported much higher frequencies. It seems that this difference is due to the genetic factors or lower exposure to cats in the Iranian population. Sensitivity to cat fur was found in 23% of our patients with allergic rhinitis, which is comparable to a study from South Africa.^26^

The present study demonstrated a 33% prevalence rate of positive SPT to DP and 36% to DF in patients with allergic rhinitis. The Mesdaghi et al.^27^ study showed a 25% prevalence rate of sensitivity to DP, and Dowaisan et al.^28^ and Seedat et al.^26^ reported sensitivity of 32.4% and 34% to DF among the same type of patients.

In allergic rhinitis patients, a 32.8% rate of positive SPT to *Alternaria* *alternata* was reported in the Iranian city of Mashhad,^29^ which chimes in with our study (28%).

Cockroach sensitivity among all the patients in our study was estimated at 18%. Approximately similar results were reported in two of Iran’s Arab neighboring countries: 19.2% in the city of Riyadh (139 patients with airway allergy)^30^ and 22.7% in Oman (689 patients).^31^ In contrast, a prevalence rate of 2.8% sensitivity to cockroaches was reported from Turkey;^32^ this finding is different from the rate reported in our country (2.8% versus 18%). These discrepancies require further research.

In our patients with allergic rhinitis, 8.4% had a positive SPT to feather mix. This finding is in accordance with studies conducted in Iran^27^ and South Africa,^26^ whose results revealed 9.5% and 10% prevalence rates of sensitivity to feather allergen.

A positive SPT to cow’s milk was seen in 21.7% of our patients. This finding is in agreement with that (21.46%) of the Khazaei et al.^33^study, which was done in the Iranian city of Zahedan.

In this study, walnuts were a common food allergen with a prevalence rate of 17.1%, which is similar to the rate reported by another study^34^ in Iran. Such similarity regarding a positive SPT to hazelnuts is seen between our study (14.9%) and another study^33^ carried out in Iran (15.32%). It is probable that the consumption of nuts is high in Iran.

In concordance with some previous studies,^35^^-^^37^ sensitivity to allergens in our patients with a positive family history (59.3%) was higher than that in those without a family history.

## Conclusion

Determination of the most common allergens and the relationship between the results of the SPT and allergic diseases in each area plays an important role in the diagnosis and management of allergic disorders and possibility of performing immunotherapy. In this study, tree mix, *Alternaria* alternate, and weed mix comprised the most common aeroallergens and milk, eggs, and wheat constituted the most common food allergens. In light of the findings of the present study, it can be concluded that appropriate preventive strategies can decrease the cost and morbidity of therapeutic measures.
